# Increased Prevalence of Pulmonary Tuberculosis in Male Adults of Sahariya Tribe of India: A Revised Survey

**DOI:** 10.4103/0970-0218.66887

**Published:** 2010-04

**Authors:** PR Sharma, Sanjay Jain, RNK Bamezai, PK Tiwari

**Affiliations:** 1 Centre for Genomics, School of Studies in Zoology (CG-SSZ), Jiwaji University, Gwalior; Jawaharlal Nehru University (JNU), New Delhi, India; 2 District Hospital, Sheopur, M. P., Jawaharlal Nehru University (JNU), New Delhi, India; 3 National Centre for Applied Human Genetics (NCAHG), SLS, Jawaharlal Nehru University (JNU), New Delhi, India

**Keywords:** Bhil, prevalence, Sahariya, sputum smear, tuberculosis

## Abstract

**Background:**

A survey made in 1991-92, reported Sahariya, a primitive tribe of India (M. P.), having high prevalence of pulmonary tuberculosis. No follow-up study was undertaken thereafter.

**Objective:**

The present study was aimed to know the current status of tuberculosis (TB) in Sahariya after more than a decade of the last survey of 1991-92, as compared to that in Bhil, another primitive tribe living in the same area but never investigated for TB incidence.

**Materials and Methods:**

A total of 763 random sputum smears from Sahariya and 169 sputum smears from *Bhil* were screened for the presence of *Mycobacterium tuberculosis* (*M..tb*) in order to evaluate the prevalence of pulmonary tuberculosis in both the tribes. Chi square (χ^2^) statistics was performed to study the correlation between age, sex on the one hand and with the prevalence of smear-positive pulmonary TB on the other hand, if any.

**Results:**

In Sahariya, the prevalence of smear-positive pulmonary TB was found increased significantly (*P*<0.005) to 0.454 as against 0.274 estimated in the earlier survey (1991-92). Males, particularly, appeared most affected (*P*<0.005; 0.382), especially adults (0.260). In contrast, among Bhil, the prevalence was very low.

**Conclusion:**

The observed increase in TB prevalence and its gender bias in Sahariya tribe indicate the high incidence rate and faster transmission of infection, especially in male sex.

## Introduction

Till date, tuberculosis (TB), caused by *Mycobacterium tuberculosis*, has remained a major health problem world over. It retains its foothold among populations, especially those with weak immunity or those living in poor socio-economic conditions. Despite implementation of effective control programs, it has remained a major cause of morbidity and mortality in all age groups of human populations around the world.(1) According to World Health Organization (WHO) data, there are about 9.27 million new cases of tuberculosis worldwide, with about 2 million people from India alone.(2)

Indian population is composed of people of diverse cultural, linguistic, biological, ethnic and genetic backgrounds, living in different socio-cultural and socio-economic settings. Madhya Pradesh, a state in central India, is a home to more than 50 tribes, most of which have been given the status of Primitive Tribal Groups (PTGs) by the Government of India. The health status of these tribes is extremely poor due to malnutrition, lack of proper hygiene and illiteracy. Lack of proper nutrition, especially protein-deficient diet in children, very often predisposes them to diseases like Marasmus and Kwashiorkor. Tuberculosis and scabies infestation were recently reported among Sahariya children.(3,4) The latest community-based cross-sectional study on tuberculosis (TB) prevalence in tribal and non-tribal populations of 11 districts of Madhya Pradesh reported that the situation of TB in the tribes of this region was not very much different from that in the non-tribal populations of the country.(5) However, the study pointed out that the risk of TB is comparatively high in tribal populations, with a noticeable gender bias in the incidence and prevalence, being higher in males than in females.

Sahariya is a primitive tribal group of North-Western Madhya Pradesh, India, reported to have high prevalence of tuberculosis.(6,7) The tribe lives in many (about 150) villages, scattered in an area of about 35-40 sq. kms, isolated from the main urban area, and with a current population of about 500,000 (census 2007 as per collector’s office, Sheopur district). Bhil, a migrant tribe from Jhabua, South of Madhya Pradesh, which is the other tribal groups, scattered in about 15 villages, with an approximate population of 100,000 (census 2007 as per collector’s office, Sheopur district), lives in close vicinity of Sahariya. But unlike Sahariya, incidence of TB is very low among Bhil.

As per the census of 1991,(8) the total population of Sahariya in the state was 417,171, of which about 350,000 were residing in the Sheopur-Gwalior region, screened for TB in the earlier survey.(9) The population of the 30 tribal villages covered in the present study is around 500,000 (census 2007 as per collector’s office, Sheopur district). The first ever survey made during December 1991 to June 1992 by Regional Medical Research Centre (RMRC), Jabalpur, M. P., India, reported a significantly high rate of sputum positivity among tribal populations as compared to their non-tribal neighbors residing in nearby areas. Since then, no comprehensive survey was made to reassess the status of tuberculosis among this tribe, even after several years (since 2004) of induction of RNTCP-DOTS (Revised National Tuberculosis Control Program-Directly Observed Treatment Short Course) program of the Government of India. The present study was aimed to document the current status of smear-positive pulmonary TB and associated risk factors among the Sahariya and Bhil tribes.

## Materials and Methods

The first survey of Sahariya tribe for TB prevalence was made in Karahal block of Morena district of M. P. state (during 1991-92, now in Sheopur district, M. P.), covering about 37 tribal villages. In the present study, 763 sputum specimens from Sahariya and 169 sputum specimens from Bhil were collected in 30-mL wide-mouth sputum containers with lid (Himedia) in a house-to-house sampling program conducted in 30 Sahariya and 5 Bhil tribal villages. The specimens were transported to field laboratory within 5-6 hours, followed by smear preparation. Two consecutive sputum samples were collected from the symptomatic persons (subjects having recurrent cough, sudden weight loss, chest pain, mild fever, etc.), as per WHO recommendations, followed by Ziehl-Neelsen’s staining for microscopic examination of acid-fast bacilli (AFB) in sputum smears (RNTCP, 2006). The tuberculosis prevalence was calculated on the basis of minimum two-time sputum-smear positivity by Ziehl-Neelsen’s method.(10) Since there were no or very few children (below 9 years) found symptomatic for TB in our random sampling, no PPD (purified protein derivative) test or Mantoux test was conducted. The earlier study was restricted only to Karahal block of Morena district of that time (1991-92), but our present (2006-07) revised survey covered many other tribal villages along with those of Karahal block. The prevalence was calculated on the basis of total number of sputum-positive samples in the entire Sahariya population in that region during the past (96/436) and the present (227/763) survey.

A well-informed and written consent was obtained from each participant. The sampling method and experimental protocols were approved by the Institutional Ethics Committee of Jiwaji University, Gwalior. Tribal subjects taking antitubercular chemotherapy (DOTS) were also enrolled for the study. The prevalence (P) was calculated by the formula(11)

$$\frac{\mathrm{Total}\;\mathrm{number}\;\mathrm{of}\;\mathrm{smear}\;-\;\mathrm{posititve}\;\mathrm{cases}\;\times \;1000}{\mathrm{Total}\;\mathrm{population}\;\mathrm{of}\;\mathrm{the}\;\mathrm{tribe}\;\mathrm{in}\;\mathrm{the}\;\mathrm{district}}$$

The Pearson’s χ^2^ statistics was performed to understand the association of sex and age with the smear-positive prevalence, if any.

## Results

### Sahariya tribe

**Figure 1:**
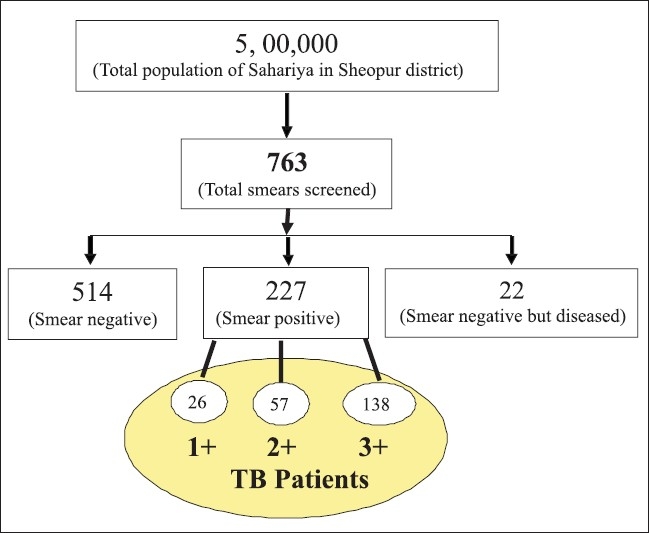
Flow chart showing sample types and categories

Sputum smears were categorized as 1+, 2+ and 3+ on the basis of number of bacilli seen per oil immersion field (as per RNTCP, WHO protocol). The smears were recorded negative, with no mycobacterium seen at all; sputum 1+ (*n*=26), with 10-99 AFB seen per 100 oil immersion fields; sputum 2+ (*n*=57), with 1-10 mycobacteria per oil immersion field (counted in 50 fields per slide); and sputum 3+ (*n*=138) with 10 or more mycobacteria per oil immersion field (counted in 20 fields per slide)(12) [Figure 1]. Six sputum smears were recorded as scanty (having rare bacilli). Mostly, adults 20-40 years of age were having disease symptoms. About 12.1% male (*n*=99) and 4.9% female (*n*=38) sputum-positive subjects were in the age group of 20-40 years, and the remaining 9.8% male (*n*=75) and 1.8% female (*n*=14) sputum-positive subjects were in all other age groups [Figure 1].

About 29.7% smears were found positive for the presence of bacilli (*M. tuberculosis*), collected from various tribal villages. Thus, the overall prevalence of smear-positive pulmonary TB was estimated to be 0.454 in the tribe [see Table 1]. The prevalence of smear-positive pulmonary TB estimated for males was found to be 0.382, which was significantly (*P*<0.005) higher, especially in the adult age group of 20-40 years (0.26), than that of females, which was 0.142 only. In the present study, the prevalence was calculated only for sputum-positive cases, excluding sputum-negative extra-pulmonary and culture-positive TB patients.

**Table 1:** Comparison of TB prevalence between the past (1991-92) and present (2006-07) studies in the Sahariya and Bhil tribes

Year of study	Villages sampled	Smear examined	Sputum positive Cases (1+, 2+, 3+)	Prevalence of smear positive pulmonary TB (no. of positive cases/population size × 1000)	*P* value
Previous study during the year 1991‐1992(6)	37	436	96	0.274	<0.005
Present study during the year 2006-07	30	763	227	0.454	
Age group					
0‐14		05	01	0.002	
15‐24		99	35	0.07	
25‐34		232	72	0.144	
35‐44		217	59	0.118	<0.005
45‐54		138	39	0.078	
55‐64		64	19	0.038	
>65		08	02	0.004	
Sex					
Male		463	191	0.382	
Female		300	71	0.142	<0.005
Bhil	05	169	4	0.04	

### Bhil

Unlike Sahariya, only 4 out of 169 random sputum samples were found positive for acid-fast bacilli after Ziehl-Neelsen’s staining and microscopy. Two of them were already taking anti-TB chemotherapy from the nearby RNTCP center.

## Discussion

Several earlier studies investigating the relevance of age and sex to the prevalence and incidence of TB(13) observed that prevalence rises with age in both the sexes.(11) The increased prevalence of smear-positive TB may likely be due to pooling of inadequately treated cases along with fresh infections. Our revised survey of Sahariya tribe revealed that despite ongoing RNTCP-DOTS program, prevalence of smear-positive pulmonary TB had increased, mainly targeting the male sex of the most productive age group of 20-40 years.

Kaulagekar and Radkar(14) highlighted the impact of socio-economic backgrounds on the prevalence, diagnosis and treatment of TB. Similar is the case of Sahariya, where extremely poor socio-economic condition-directed malnutrition background becomes one of the main factors predisposing to TB. We found Bhils to be comparatively better in all aspects, including health, education, lifestyle, health-care-seeking behavior, etc. Most likely, the above factors could be mainly associated with the high prevalence of smear-positive pulmonary TB in Sahariya and low prevalence in Bhil, while both have been given primitive tribal status. TB prevalence data on Sahariya also revealed a gender bias, indicating males of Sahariya tribe to be at higher risk of infection than females. Apart from possible biological reasons, such as differences between males and females in thresholds of innate immunity to fight infections, there may be several other factors associated with the observed gender bias in case of TB prevalence. One such common factor seen in the earlier as well as in the present study is the health-care-seeking behavioral difference in male and female sexes. During house-to-house sampling in Sahariya tribe, it was observed that females of Sahariya tribe generally hide the disease symptoms and remain indoors for most of the time, being found least interested in expressing themselves before the investigating team and thus escape active (house-to-house search of symptomatic subjects) as well as passive (OPD in hospital) medical attention, which could be a likely reason for very low prevalence of smear positivity among females.

The data on tuberculosis prevalence from SAARC countries,(15) such as Bangladesh (*P*= 0.03), India (*P*<0.0001), Nepal (*P*<0.0001) and Sri Lanka (*P*= 0.01), have also revealed high sputum positivity among male TB suspects than among female TB suspects.

Several studies have explored reasons behind the gender bias in tuberculosis susceptibility and found that fear and stigma associated with TB makes greater impact on women than on men.(16) The analysis of epidemiological data by Thorson *et al*.(17) also reflects gender to have an impact on the disease and its control. It seems, therefore, that the observed differences in disease prevalence in males and females may or may not have any direct biological basis; however, they are strongly associated with malpractice of health care policies by care providers or care seekers.

The global data on tuberculosis prevalence have shown that the prevalence of *M. tb* infection is similar in males and females until adolescence; but after that, it appears higher in males.(18) In fact, women cases often remain under-notified to the public health authorities, if relying on passive case-findings only. It is therefore essential for both health care providers and patients to strengthen the awareness program about TB and improve accessibility of health care services under a vertical control strategy. (19) The National Tuberculosis Control Programme (NTCP) should also supervise and assess the possible gender bias, if any, and reasons behind it should be investigated thoroughly, at least, among at-risk communities. This is essential, because an undiagnosed female host may serve as a potential source of latent infection, especially in communities like Sahariya tribe, who live in small houses in close proximities of each other, with likely chances of further transmission of *M. tb* infection.

Our data indicates that the status of TB in Sahariya appeared maintained even after more than a decade. It is very much consistent with no or only elementary improvement in the nutritional status and general health of the Sahariya people, which hints at its close association with the prevailing demographic conditions. Among the most likely risk factors that make the male sex of young age group to be at higher risk of developing tuberculosis with a faster rate of transmission of the disease are group smoking and alcohol abuse during working hours. HIV infection, diabetes, etc., the other common risk factors reported in association with TB in many studies, however, have not been investigated yet. Further, many of the young Sahariya men also work in stone mines, where they develop silicosis, which later precipitates into clinically active disease and ultimately death. On the other hand, females, who generally remain indoors (less health-care-seeking behavior) for most of the time, escape these occupational hazards to a larger extent, which could be one of the likely reasons for low prevalence of smear positivity among them. Our present estimate of smear-positive pulmonary TB in Sahariya thus supports the findings of the earlier study, which observed the rate of pulmonary tuberculosis to be significantly (*P*<0.05) higher among males than in females.(6)

Despite its limitations, like prevalence calculated only on the basis of sputum smear positivity and non-ascertainment of denominator, the present study is significant in view of the current global status of the disease and its likely elevation to MDR (multidrug-resistant) and XDR (extremely drug resistant) status. It stresses the need of targeted strategies with even more improved diagnosis, treatment and prevention programs, particularly in at-risk groups and those living in rural and remote areas, who generally remain neglected due to inaccessibility. Also, there is a need to give more emphasis to education in order to make them aware of common risk factors and importance of adherence to the 6-month-long anti-TB chemotherapy, which they generally discontinue prematurely, resulting in a still worse disease condition. The possible reasons behind gender bias in TB prevalence in Sahariya must also be investigated in a more comprehensive way in order to get a clear picture of prevalence of M. tb infection in the two sexes. Therefore, in addition to ongoing TB control programs, more stringent strategies should be designed and launched through regular active (house-to-house) and passive (OPD at hospitals) diagnosis and treatment of tuberculosis in such communities to control the menace, even if complete eradication is not possible.
